# Antimicrobial Materials with Medical Applications

**DOI:** 10.3390/ijms23031890

**Published:** 2022-02-08

**Authors:** Christina N. Banti, Sotiris K. Hadjikakou

**Affiliations:** Laboratory of Biological Inorganic Chemistry, Department of Chemistry, University of Ioannina, 45110 Ioannina, Greece; cbanti@uoi.gr

This Special Issue of the *International Journal of Molecular Sciences*, entitled “Antimicrobial Materials with Medical Applications”, covers a selection of recent research and review articles in the field of antimicrobial materials, as well as their medical applications. Moreover, it provides an overview of recent developments and the latest research in this increasingly diverse field. It also presents, with particular emphasis, the applications of new antimicrobial surfaces, medical devices, contact lens, packaging materials, etc.

Infectious diseases are a continuous threat to human health. New methods for the appropriate use of disinfectants and antibiotics have been developed to reduce microbial activity, associated infections, and increases in antimicrobial resistance. To overcome microbial infections and antimicrobial resistance, various antimicrobial materials, including small molecules and macromolecules, and inorganic and organic agents, have been developed and evaluated. Thus, the healthcare sector is facing totally new challenges. Potential and promising weapons against bacterial growth and the development of multi-drug resistant bacteria have been found in new antimicrobial materials. The development of new long-term or permanent antimicrobial materials, which go beyond the resistance of microbes to modern antibiotics is a research, technological, and financial issue of great importance. During the preparation of this Special Issue, the current worldwide public health crisis of COVID-19 particularly highlighted the emergent need for materials that inactivate on contact, not only microbes, but also viruses, further emphasizing the importance of the development of new antimicrobial and antiviral materials.

This Special Issue is composed of thirteen articles that are briefly reviewed below.

Jain et al. reviewed recent advances in green synthesis, in the context of the physicochemical and biological properties of green silver nanoparticles [1]. Coelho et al. reviewed the effects of different cavity disinfectants on bond strength and the clinical success of composites and glass ionomer restorations of primary teeth [2]. van Hengel et al. presented a review on the biomaterial properties, antibacterial behavior, and biocompatibility of titanium implants that were biofunctionalized by plasma electrolytic oxidation (PEO) using Ag, Cu, and Zn [3]. Coelho et al. conducted a review on the effects of different cavity disinfectants on restoration adhesion and clinical success [4]. Meretoudi et al. dispersed silver nanoparticles (AgNPs(ORLE)) of oregano leaf extract (ORLE) in polymer hydrogels (pHEMA@ORLE_2 and pHEMA@AgNPs(ORLE)_2) using hydroxyethyl–methacrylate (HEMA). The materials were characterized and the antimicrobial activity of the materials was investigated against Gram-negative or Gram-positive bacteria strains [5]. Hung et al. synthesized curcumin analogs, and tested their antibacterial activity against Gram-positive aerobic bacteria [6]. Bidossi et al. investigated the in vitro ability of antibiotic-eluting hydroxyapatite/calcium sulfate bone graft substitute to prevent bacterial adhesion and biofilm formation by clinically relevant microorganisms [7]. Piszczek et al. assessed the microbiocidal activity of tri- and tetranuclear oxo-titanium(IV) complexes, which were dispersed in a poly(methyl methacrylate) matrix [8]. Marinas et al. extracted and characterized cellulose from *Gleditsia triacanthos* pods, and used it to fabricate a wound dressing. Moreover, the antioxidant properties and the antimicrobial activities of these materials were evaluated [9]. Li et al. proposed a simple and eco-friendly strategy to efficiently assemble zinc oxide nanoparticles and silver nanoparticles on sericin–agarose composite film to impart superior antimicrobial activity [10]. Khan et al. presented the green synthesis of chromium oxide nanoparticles using a leaf extract of *Abutilon indicum (L.) Sweet* as a reducing and capping agent. The biological activities were also evaluated [11]. Gouyau et al. prepared, synthesized, and tested the antibacterial activity of 12 nm gold and silver nanoparticles [12]. Tuñón-Molina et al. developed a single-use transparent antimicrobial face shield composed of polyethylene terephthalate and an antimicrobial coating of benzalkonium chloride for facial protective equipment [13].
